# Correction to ‘A Brief Cognitive Analytic Therapy‐Informed Approach for Young People That Have Self‐Injured (CATCH‐Y): A Case Series’

**DOI:** 10.1002/cpp.70054

**Published:** 2025-03-06

**Keywords:** cognitive analytic therapy, non‐suicidal self‐injury, self‐harm, young people

Haw, R. , Marsden, M. , Hartley, S. , Turpin, C. and Taylor, P. 2024. “A Brief Cognitive Analytic Therapy–Informed Approach for Young People That Have Self‐Injured (CATCH‐Y): A Case Series.” Clinical Psychology and Psychotherapy 31: e2976. 10.1002/cpp.2976.38757462

In the abstract under the ‘Results’ section it reads as follows:

‘Measures showed preliminary support for positive change in rates of NSSI, urges to self‐harm, low mood and personal recovery, although results were mixed.’

This is an error and this sentence should read as follows:

‘Measures showed preliminary support for positive change in rates of low mood and personal recovery, although results were mixed.’

We apologise for this error.

